# Evaluating the prevalence of somatization symptoms in the family of cancer patients

**DOI:** 10.1038/s41598-025-85474-5

**Published:** 2025-07-02

**Authors:** Mohammad Reza Sharbafchi, Fateme Naji, Soleyman Ghaderi, Mahboube Aghanouri, Zahra Barouti

**Affiliations:** 1 Behavioral Sciences Research Center, Department of Psychiatry, Isfahan University of Medical Sciences, Iranian Cancer Control Center (MACSA), Isfahan, Iran; 2 Behavioral Sciences Research Center, Department of Psychiatry, School of Medicine, Isfahan University of Medical Sciences, Isfahan, Iran; 3 Clinical Psychologist, Behavioral Sciences Research Center, Isfahan University of Medical Sciences, Isfahan, Iran

**Keywords:** Psycho-oncology, Somatization symptoms, Relatives, Family members, Breast neoplasms, Colon neoplasms, Lung neoplasms, Cancer, Psychology, Oncology

## Abstract

The psychological well-being of family members caring for cancer patients impacts care and the health of both parties. This study examines somatization symptoms prevalence among families of breast, lung, and colorectal cancer patients. This cross-sectional study at Omid Hospital and Isfahan Cancer Prevention and Control Center (September 2018 to September 2019) randomly involved 780 family caregivers. Demographic data and the patient’s medical history were collected. Somatic symptoms were assessed using the Screening for somatoform symptoms and Patient Health Questionnaire. Statistical analysis used SPSS version 23, with significance at *P* < 0.05. Among 780 subjects (mean age: 40.56 ± 13.88 years), 407 (52.2%), 317 (40.6%), and 56 (7.2%) cared for colorectal, breast, and lung cancer patients, respectively. Caregivers of lung cancer patients reported significantly more excessive thoughts, feelings, or behaviors related to somatic symptoms (*P* = 0.027) along with neurological, cardio-respiratory, gastrointestinal, urogenital, and musculoskeletal symptoms (*P* < 0.001). General complaints were more prevalent among relatives of colorectal cancer patients (*P* < 0.001). 89.5% of caregivers exhibited excessive somatic symptom-related thoughts, feelings, or behaviors significantly associated with their relationship level with the cancer patient (*P* = 0.003). Caregivers of lung cancer patients (*P* = 0.028), female (*P* = 0.001), married (*P* = 0.005), and unemployed individuals (*P* = 0.001) reported higher PHQ-15 scores (mean score = 8.53 ± 5.73) indicating more frequent moderate to severe symptoms. Symptom severity showed no considerable association with age, education, patient illness duration, and relation level with the patient. Somatization symptoms are prevalent among caregivers of cancer patients. Targeted interventions, such as mental health screening and psychosocial support, are crucial to address caregivers’ psychological well-being.

## Introduction

Cancer incidence rates have increased over the past 30 years, becoming a main global health issue. While mortality rates have decreased, cancer remains the second cause of death worldwide, expected to surpass heart disease by 2060. The most common cancers vary by gender, with lung cancer being the cause of cancer-related death in both men and women^1–4^.

In recent years, the growth in cancer cases, coupled with improvements in patient survival and preferences for receiving outpatient care, has highlighted the vital role of cancer patient’s families as caregivers. However, taking care of cancer patients can be a daunting and involuntary responsibility, leading to physical, emotional, social, or financial challenges for caregivers^5–7^. Studies have declared this fact that cancer imposes a massive psychological burden on family caregivers, and this burden increases with the intensity of the care provided. The distress faced by cancer patient family members can manifest as somatic symptoms and other psychological problems like depression and anxiety, and these psychological consequences can persist long after the end of caregiving, highlighting the need for ongoing support^5,8–11^. The interdependence of the psychological well-being of cancer patients and their loved ones cannot be denied. For instance, research indicates that feelings of depression and anxiety experienced by cancer patients are closely linked to similar experiences by their family members who care for them, resulting in increased levels of distress and somatization^12^. These mental consequences may be influenced by several factors, such as the caregiver’s sex, age, education, income, underlying psychiatric disorders, attitudes towards illness, their patient’s cancer stage, and availability of supportive services^5,13–15^.

The physical and mental health of cancer patients and their caregivers are linked. Caregivers who were mentally and physically healthy could better devote themselves to patient care^8,12,16–18^.

While the literature has explored the psychological impact of cancer on caregivers, there is a limited understanding of the specific prevalence and factors associated with somatization symptoms in this population. This study aims to fill this gap by evaluating the prevalence of somatization symptoms among caregivers of breast, lung, and colorectal cancer patients and identifying potential risk factors.

## Materials and methods

This cross-sectional study was performed at the Omid Hospital and Iranian Cancer Control Center (MACSA) in Isfahan over one year from September 2018 to September 2019. MACSA is a referral cancer institute that provides supportive and palliative care to cancer patients and their families.

### Participants

A simple random sampling method was used to select first-degree relatives (*n* = 780) caring for patients aged 18–65 with breast, lung, or colon cancer. Patients could be in any stage of cancer up to 6 months post-treatment. Their patient could be in any stage of cancer up to 6 months after receiving curable treatment. Exclusion criteria included illiteracy, underlying medical diseases, pregnancy, breastfeeding, refusal to participate, and diagnoses of anxiety, somatic symptoms, or psychotic disorders based on DSM-V criteria. Also, the researchers conducted interviews with the participants and excluded any cases diagnosed with anxiety, somatic symptoms, and psychotic disorders based on DSM-V diagnostic criteria.

The sample size was calculated using a power analysis with a significance level of 0.05, a power of 0.80, and an expected effect size of 0.30. This analysis determined a minimum sample size of 750 participants.

### Ethical considerations

This research followed the Declaration of Helsinki on Biomedical Research Involving Human Subjects^19^. The Medical Research Ethics Committee of Isfahan Medical University has approved it (Approval code: IR.MUI.RESEARCH.REC.1398.484). In the informed consent form, we provided participants with comprehensive information about the study’s aims and the questionnaires’ instructions. The participants were assured of the confidentiality of their data. Hence, after reading the informed consent form, all ambiguities were thoroughly explained to them. Participants who chose to cooperate with us signed the consent form.

### Data collection

In the present study, demographic information, including age, sex, marriage status, education level, occupation, relation level with patient, their patient cancer type, and duration, were collected. Two standard questionnaires, which are mentioned below, are used to assess somatic symptoms:

1. The Screening for somatic symptom disorders-7 (SOMS-7).
2. The Patient Health Questionnaire-15 (PHQ-15).

#### Screening for somatic symptom disorders-7 (SOMS-7)

This scale assesses two aspects of somatic symptoms: presence and severity. It consists of 53 somatic symptoms based on the DSM-IV and ICD-10 list of physical and autonomic somatic symptoms. Forty-seven symptoms are shared between the two sexes; 5 are specialized for females and one for males. Each item is answered YES if they experience these symptoms without any medical reason diagnosed by physicians, and NO if they have not. Also, if the severity of symptoms is evident, respondents rate the severity of each item using a 5-point Likert scale: 0-not at all to 4 very severe. Following that, items 54 to 68 specifically are related to DSM-IV and ICD-10 exclusion and inclusion criteria for somatic symptom disorders^20^. Rief and Hiller confirmed the high reliability and sensitivity of SOMS-7 in evaluating the treatment efficacy in somatization symptoms^21^. The Persian version of this questionnaire is available with high internal consistency. The Cronbach alpha for the healthy population and patients with somatic symptoms was 0.94 and 0.92, respectively^22^.

#### The patient health questionnaire-15

Another tool used to evaluate the somatization is the Patient Health Questionnaire-15. This self-reported questionnaire includes 15 common somatic symptoms, and respondents rate each item’s severity using a three-point Likert scale: 0 = not bothering at all, 1 = bothering a little, and 2 = bothering a lot. Each item score is summed up to achieve a total score ranging from 0 to 30. According to a total score, the severity of symptoms is classified into four categories: minimum (score ≤ 4), mild (5 ≤ scores ≤ 9), moderate (10 ≤ scores ≤ 14), and severe (scores ≥ 15). The Persian version of this questionnaire is available with acceptable internal reliability (α = 0.76), too^23–25^.

### Statistical analysis

The continuous quantitative variables are reported as means (± standard deviation (SD)) and categorical variables as numbers (percentages). The normality of the data is determined graphically and statistically using Kolmogorov–Smirnov test. The ANOVA test compares the mean age between cancer types and the PHQ-15 symptom severity category. In addition, the Chi-square test is used to evaluate the association of somatic symptoms presence (SOMS-7) and severity (PHQ-15) with the independent variables: sex, the participants’ patient cancer type and duration, relation level with patient, marriage status, education level, occupation. To summarize the results of SOMS-7 analysis, the somatic symptoms of SOMS-7 are classified into seven groups: cardio-respiratory, neurological, gastrointestinal, urogenital, musculoskeletal, and general symptoms (see Table 1). A P-value < 0.05 is considered statistically significant.

**Table 1:** Details of SOMS-7 items classification.

General	Loss of appetite, Dry mouth, Sweating, Flushing or blushing, Excessive tiredness upon mild exertion, Blotchiness or discoloration of the skin
Neurological	Headaches, Impaired coordination or balance, Memory loss, Paralysis or localized weakness, Unpleasant numbness or tingling sensations, Double vision, Hearing loss, Blindness, Aphonia (loss of voice), Loss of touch or pain sensations, Hallucinations, Loss of consciousness, Seizures
Cardio-Respiratory	Chest pain, Heart palpitations, Breathlessness without exertion, Painful breathing, or hyperventilation
Gastrointestinal	Abdominal Pain, Anal Pain, Nausea, Bloating, Discomfort in and around the precordium, Vomiting (excluding pregnancy), Regurgitation of food, Hiccoughing or burning sensation in chest or stomach, Food intolerance, Bad taste in the mouth or excessively coated tongue, Frequent diarrhea, Discharge of fluid from the anus, Frequent bowel movements, Stomach discomfort or churning feeling in the stomach, Difficulty swallowing or lump in the throat,
Urogenital	Pain during sexual intercourse, Pain during urination, Frequent urination, lack of sexual desire (loss of libido), Unpleasant sensations inside or around the genitalia, Urinary retention For women only: Painful menstruation, Irregular menstruation, Excessive menstrual bleeding, Continuous or frequent vomiting during pregnancy, Unusual or copious vaginal Discharge For men only: Erectile or ejaculatory dysfunction
Musculoskeletal	Back Pain, Joint Pain, Pain in legs and arms
Excessive thoughts, feelings, or behaviors related to somatic symptoms	Items 54–68 of SOMS-7 questionnaire ^(21)^

## Result

Our study included 780 participants comprising 407 (52.2%), 317 (40.6%), and 56 (7.2%) individuals who had caretaking responsibilities for patients with colorectal, breast, and lung cancer, respectively. The participants had a mean age of 40.56 ± 13.88 years (with a range of 14–83 years), and 56.5% were women. Table 2 provides a summary of all characteristics of our participants based on their patient cancer type.

**Table 2:** Participants’ characteristics by their patient cancer type.

Variables		Total	Type of cancer			*P*-value
			Breast 317 (40.6)	Colorectal 407 (52.2)	Lung 56 (7.2)	
Age Mean ± SD		40.56 ± 13.88	40.26 ± 14.58	40.57 ± 13.47	42.21 ± 12.80	0.56
Sex N (%)	Female	441 (56.5)	179 (56.5%)	228 (56.0%)	34 (60.7%)	0.801
	Male	339 (43.5)	138 (43.5%)	179 (44.0%)	22 (39.3%)	
Marriage status N (%)	Single	218 (27.9)	105 (33.1%)	102 (25.1%)	11 (19.6%)	**0.020**
	Married	562 (72.1)	212 (66.9%)	305 (74.9%)	45 (80.4%)	
Education level N (%)	Primary education	266 (34.1)	107 (33.8%)	137 (33.7%)	22 (39.3%)	0.942
	High school diploma	284 (36.4)	111 (35.0%)	153 (37.6%)	20 (35.7%)	
	Under-graduate	190 (24.4)	82 (25.9%)	97 (23.8%)	11 (19.6%)	
	Post-graduate or higher	40 (5.1)	17 (5.4%)	20 (4.9%)	3 (5.4%)	
Occupation N (%)	Unemployed	336 (43.1)	118 (37.2%)	193 (47.4%)	25 (44.6%)	**0.008**
	Employee	168 (21.5)	63 (19.9%)	93 (22.9%)	12 (21.4%)	
	Self-employed	276 (35.4)	136 (42.9%)	121 (29.7%)	19 (33.9%)	
Relation with patient N (%)	Child	277 (35.5)	135 (42.6%)	118 (29.0%)	24 (42.9%)	**0.000**
	Parent	267 (34.2)	59 (18.6%)	194 (47.7%)	14 (25.0%)	
	Partner	141 (18.1)	64 (20.2%)	62 (15.2%)	15 (26.8%)	
	Sibling	95 (12.2)	59 (18.6%)	33 (8.1%)	3 (5.4%)	
Cancer duration N (%)	One month to 1 year	146 (18.7)	60 (18.9%)	70 (17.2%)	16(28.6%)	0.122
	One year to 5 years	634 (81.3)	257 (81.1%)	337 (82.8%)	40(71.4%)	

Bold values indicate statistical significance (*P* < 0.05).

Table 3 shows the association between patients’ cancer type and their relatives’ prevalence of somatic symptoms. The study found that the manifestation of all neurological, cardio-respiratory, gastrointestinal, urogenital, and musculoskeletal symptoms was significantly higher in the relatives of patients with lung cancer. General symptoms were more frequent in the relatives of patients with colorectal cancer. The study also highlighted that the family members of lung cancer patients had significantly higher rates of excessive thoughts, feelings, or behaviors related to somatic symptoms.

**Table 3:** The somatic symptoms distributions according to the cancer type of participants patient (SOMS-7 data):

SOMS-7 Somatic symptoms *N* (%)	Type of cancer			*P*-value
	Breast 317 (40.6)	Colorectal 407 (52.2)	Lung 56 (7.2)	
General	183 (57.7%)	324 (79.6%)	44 (78.6%)	**< 0.001**
Neurological	195 (61.5%)	317 (78.1%)	47 (83.9%)	**< 0.001**
Cardio-Respiratory	149 (47.0%)	281 (69.0%)	45 (80.4%)	**< 0.001**
Gastrointestinal	190 (59.9%)	335 (82.3%)	50 (89.3%)	**< 0.001**
Urogenital	170 (53.6%)	307 (75.4%)	43 (76.8%)	**< 0.001**
Musculoskeletal	178 (56.2%)	297 (73.0%)	45 (80.4%)	**< 0.001**
Excessive thoughts, feelings, or behaviors related to somatic symptoms	292 (92.1%)	352 (86.7%)	53 (94.6%)	**0.027**

Bold values indicate statistical significance (*P* < 0.05).

Table 4 lists the distribution of symptoms based on the characteristics of our subjects. The study reveals that 89.5% of participants experienced excessive thoughts, feelings, or behaviors related to somatic symptoms. Additionally, gastrointestinal complaints were the most frequent symptom (73.7%). Females were observed to have experienced all symptoms significantly more than males. Moreover, married individuals mainly presented with more cardio-respiratory, urogenital, and musculoskeletal symptoms. Unemployed subjects experienced all symptoms significantly more than employees or self-employed individuals, except for neurological symptoms. The parents or children of cancer patients were more likely to experience general, neurological, cardio-respiratory, and urogenital symptoms than other family members.

**Table 4:** The somatic symptoms distributions according to the participants’ characteristics (SOMS-7 data):

Variables *N* (%)		SOMS-7 somatic symptoms						
		General 551 (70.6)	Neurological 559 (71.8)	Cardio-Respiratory 475 (60.9)	Gastrointestinal 575 (73.7)	Urogenital 520 (66.7)	Musculoskeletal 520 (66.7)	Excessive thoughts, feelings, or behaviors related to somatic symptoms 697 (89.5)
Sex	Female	333 (75.5)	343 (78.0)	290 (65.8)	290 (65.8)	338 (76.6)	312 (70.7)	396 (90.0)
	Male	218 (64.3)	216 (63.7)	185 (54.6)	185 (54.6)	182 (53.7)	208 (61.4)	301 (88.8)
*P*-value		**0.001**	**< 0.001**	**0.002**	**0.034**	**< 0.001**	**0.006**	0.586
Marriage status	Single	145 (66.5)	152 (69.7)	115 (52.8)	156 (71.6)	128 (58.7)	132 (60.6)	193 (88.9)
	Married	406 (72.2)	407 (72.5)	360 (64.1)	419 (74.6)	392 (69.8)	388 (69.0)	504 (89.7)
*P*-value		0.115	0.124	**0.004**	0.394	**0.003**	**0.024**	0.763
Education level	Primary education	191 (71.8)	191 (71.8)	172 (64.7)	194 (72.9)	180 (67.7)	185 (69.5)	238 (89.5)
	High school diploma	204 (71.8)	206 (72.5)	169 (59.5)	218 (76.8)	196 (69.0)	188 (66.2)	256 (90.5)
	Under-graduate	127 (66.8)	135 (71.4)	110 (57.9)	136 (71.6)	119 (62.6)	124 (65.3)	167 (87.9)
	Post-graduate or higher	29 (72.5)	27 (67.5)	24 (60.0)	27 (67.5)	25 (62.5)	23 (57.5)	36 (90.0)
*P*-value		0.624	0.929	0.467	0.442	0.470	0.440	0.848
Occupation	Employee	115 (68.5)	120 (71.4)	100 (59.5)	124 (73.8)	108 (64.3)	105 (62.5)	150 (89.3)
	Unemployed	258 (76.8)	252 (75.2)	224 (66.7)	268 (79.8)	256 (76.2)	243 (72.3)	298 (89.0)
	Self-employed	178 (64.5)	187 (67.8)	151 (54.7)	183 (66.3)	156 (56.5)	172 (62.3)	249 (90.2)
*P*-value		**0.003**	0.432	**0.010**	**0.001**	**< 0.001**	**0.014**	0.876
Relation with patient	Child	186 (67.1)	195 (70.4)	152 (54.9)	194 (70.0)	175 (63.2)	172 (62.1)	257 (92.8)
	Parent	207 (77.5)	208 (78.2)	183 (68.5)	209 (78.3)	197 (73.8)	189 (70.8)	230 (86.5)
	Partner	96 (68.1)	89 (63.1)	87 (61.7)	106 (75.2)	84 (59.6)	97 (68.8)	132 (93.6)
	Sibling	62 (65.3)	67 (70.5)	53 (55.8)	66 (69.5)	64 (67.4)	62 (65.3)	78 (82.1)
*P*-value		**0.023**	**0.012**	**0.008**	0.120	**0.013**	0.171	**0.003**
Cancer duration	One month to 1 year	109 (74.7)	106 (72.6)	97 (66.4)	117 (80.1)	93 (63.7)	101 (69.2)	135 (92.5)
	One year to 5 years	442 (69.7)	453 (71.6)	378 (59.6)	458 (72.2)	427 (67.4)	419 (66.1)	562 (88.8)
*P*-value		0.237	0.802	0.128	0.051	0.399	0.47	0.191

Bold values indicate statistical significance (*P* < 0.05).

**Fig. 1:**
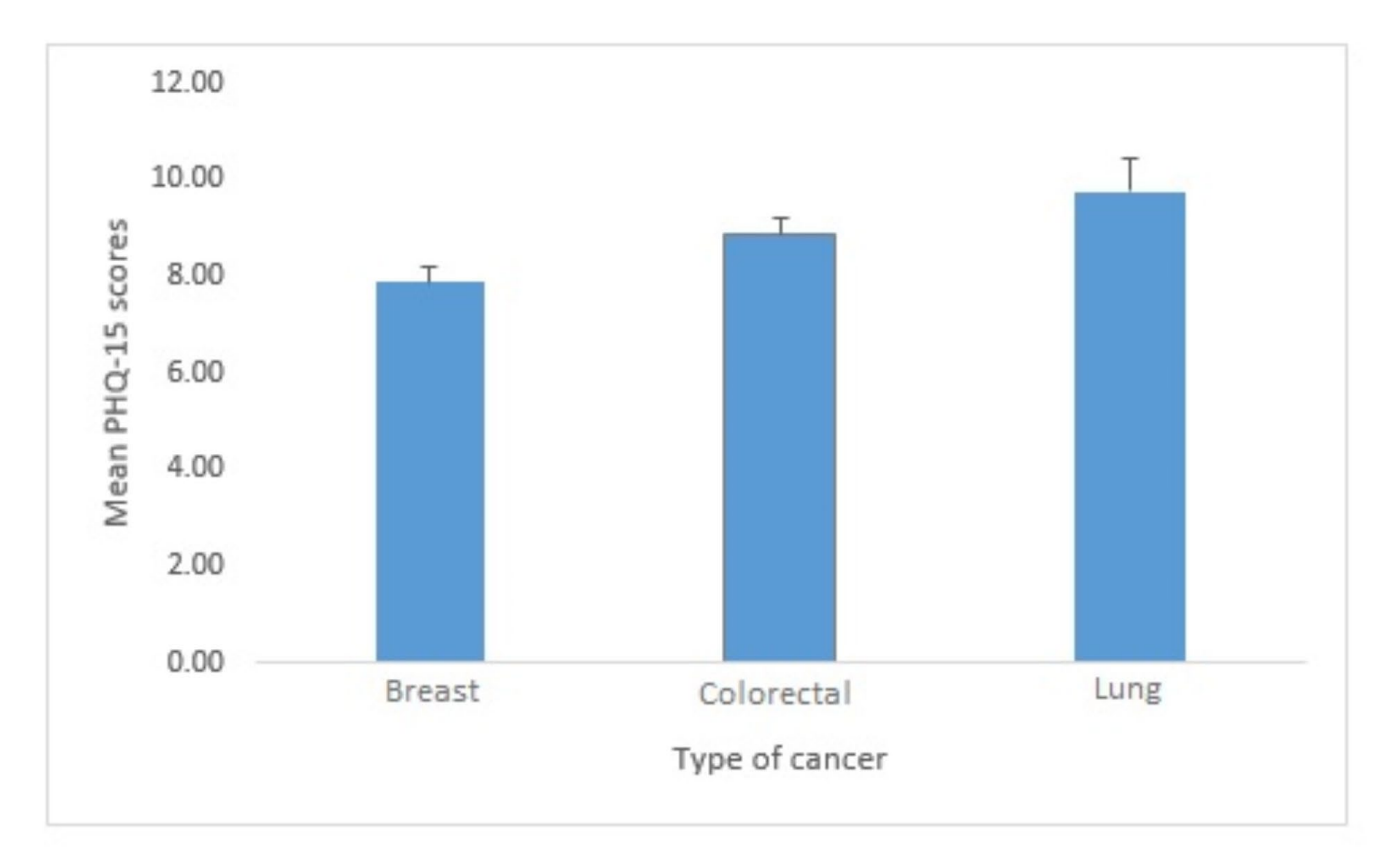
Comparison of the mean (SE) scores of PHQ-15 in the relatives of patients with breast, colorectal, and lung cancer.

The mean PHQ-15 scores were 8.53 ± 5.73, and comparing the mean scores and their standard error showed the relatives of patients with lung cancer had significantly higher scores (*P* = 0.009, See Fig. 1). Also, the study found a significant association with sex, marriage status, occupation, and cancer type. Table 5, providing detailed information, indicates females (*P* = 0.001), married individuals (*P* = 0.005), and unemployed individuals (*P* = 0.001) were significantly more likely to experience moderate to severe somatic symptoms, as measured by the PHQ-15 score. In terms of cancer type, PHQ-15 analysis revealed that caregivers of lung cancer patients were more likely to experience moderate levels of somatic symptoms. In contrast, caregivers of breast cancer patients were more likely to report minimal symptoms. Caregivers of colorectal cancer patients exhibited the highest levels of somatic symptom severity (*P* = 0.028). Nevertheless, age, educational level, duration of the illness, and relation level with the patient did not have any significant association with questionnaire scores.

**Table 5:** Association of the severity of somatic symptoms with participant’s characteristics (PHQ-15 data):

Variables *N* (%)		Minimum 217 (27.82)	Mild 224 (28.72)	Moderate 208 (26.67)	Severe 131 (16.79)	*P*-value
Sex	Female	100 (22.7)	130 (29.5)	123 (27.9)	88 (20.0)	**0.001**
	Male	117 (34.5)	94 (27.7)	85 (25.1)	43 (12.7)	
Marital status	Single	79 (36.2)	63 (28.9)	48 (22.0)	28 (12.8)	**0.005**
	Married	138 (24.6)	161 (28.6)	160 (28.5)	103 (18.3)	
Occupation	Unemployed	70 (20.8)	94 (28.0)	100 (29.8)	72 (21.4)	**0.001**
	Employee	51 (30.4)	55 (32.7)	46 (27.4)	16 (9.5)	
	Self-employed	96 (34.8)	75 (27.2)	62 (22.5)	43 (15.6)	
Type of cancer	Breast	104 (32.8)	94 (29.7)	76 (24.0)	43 (13.6)	**0.028**
	Lung	9 (16.1)	15 (26.8)	22 (39.3)	10 (17.9)	
	Colorectal	104 (25.6)	115 (28.3)	110 (27.0)	78 (19.2)	
Education level	Primary education	64 (24.1)	79 (29.7)	69 (25.9)	54 (20.3)	0.628
	High school diploma	80 (28.2)	82 (28.9)	78 (27.5)	44 (15.5)	
	Under-graduate	59 (31.1)	53 (27.9)	49 (25.8)	29 (15.3)	
	Post-graduate or higher	14 (35.0)	10 (25.0)	12 (30.0)	4 (10.0)	
Relation with patient	Child	88 (31.8)	81 (29.2)	67 (24.2)	41 (14.8)	0.279
	Parent	62 (23.2)	86 (32.2)	72 (27.0)	47 (17.6)	
	Partner	40 (28.4)	30 (21.3)	42 (29.8)	29 (20.6)	
	Sibling	88 (31.8)	81 (29.2)	67 (24.2)	41 (14.8)	
Cancer duration	One month to 1 year	30 (20.5)	48 (32.9)	42 (28.8)	26 (17.8)	0.179
	One year to 5 years	187 (29.5)	176 (27.8)	166 (26.2)	105 (16.6)	
Age Mean ± SD		39.71 (14.09)	39.85 (14.04)	41.75 (13.41)	41.02 (14.31)	0.383

Bold values indicate statistical significance (*P* < 0.05).

## Discussion

The present study demonstrates a high prevalence of somatization symptoms in individuals closely connected to cancer patients (89.5%) and suggests a potential risk for developing somatization disorder. Notably, family members dealing with lung cancer patients reported a significantly greater likelihood of experiencing these symptoms compared to those supporting individuals with breast and colorectal cancer. Amongst family members providing care for lung cancer patients, there was a high prevalence of symptoms related to various bodily systems such as neurological, cardio-respiratory, gastrointestinal, and urogenital. These caregivers also exhibited more severe symptoms compared to others. Our findings also suggest that the frequency of somatization symptoms is potentially associated with sex, relation level with cancer patients, marriage status, and occupation. Additionally, the severity of symptoms is related to cancer type, sex, marriage status, and occupation.

Recent research has consistently highlighted the significant psychological impact of cancer on caregivers. For instance, a systemic review on literature published between 2001 and 2022 showed a high prevalence of depression among cancer patients’ caregivers^26^. Another cross-sectional study on caregivers of cancer patients with cancer in Zanjan- Iran, reported a high level of depression, anxiety, and burden in this study population^27^.

In our study population, gastrointestinal symptoms were most commonly reported. A review in 2010 surveyed several studies and concluded that family caregivers experience somatic symptoms, including sleep problems, pain, fatigue, loss of appetite, weight loss, and physical weakness as the most common symptoms^17^.

We found that females experienced all symptoms significantly more frequently in our population. Also, there was a significant correlation between relation level and frequency of some somatic symptoms; Parents and children of cancer patients may experience general, neurological, cardio-respiratory, and urogenital symptoms more frequently than other family members. Additionally, we found that the frequency of symptoms varied according to marriage status and occupation. Married individuals were more likely to report cardio-respiratory, urogenital, and musculoskeletal symptoms, whereas unemployment was associated with an increased incidence of all these symptoms except for neurological symptoms. In a study involving 112 family members taking care of end-stage cancer patients, it was found that 50.9% of them had a high level of somatization. Furthermore, the study revealed that sex was a predictor factor for psychological morbidities such as depression, anxiety, distress, and somatization, with all of these symptoms being significantly more prevalent in women. These results align with our findings, however, this study did not establish any significant association between somatization and age, education, or relationship status^28^. Align with our results regarding the relation of caregivers’ sex and somatic symptom frequency, a meta-analysis exploring distress in couples affected by cancer found that females experience more distress than males due to their role in dealing with cancer^15^. Also, among 234 caregivers of patient suffering from head and neck cancer, being female was associated with more health problems^29^. Another cross-sectional study found out being female associated with higher distress level^30^. This relation resonated with a recent meta-analysis conducted on 45 published studies that evaluated the depression prevalence among caregivers of cancer patients^31^.

The findings of this study regarding the association between lung cancer and higher probability of experiencing somatic symptoms, differ from those reported by the recent Korean study report. They estimated the burden on caregivers of lung cancer patients was mild to moderate^32^.

Our analysis revealed that several demographic factors were associated with somatic symptom severity. Especially female caregivers, married individuals, and unemployed individuals were more likely to experience moderate to severe symptoms. However, there was no correlation between relation level and severity of symptoms. A cross-sectional examined the caregivers of patients under chemotherapy; the authors observed a mean somatization score of 2.51 ± 2.41. Also, an association was established between the somatization score and various factors such as age, gender, marital status, the relation level with the patient, occupation, and underlying chronic disease^33^. A cross-sectional study was conducted on 120 relatives of patients who presented at the emergency department due to any disease to evaluate their psychological symptoms. The Symptom Check List (SCL-90) was utilized for this purpose. The mean score for somatization in participants exceeded 1, with females scoring significantly higher than males^34^. This study highlights the significant prevalence of somatization symptoms among caregivers of cancer patients, emphasizing the need for increased attention to their mental health. The findings suggest that targeted interventions, such as mental health screening, psychosocial support, and education programs, are crucial to addressing caregivers’ psychological well-being.

By identifying risk factors for somatization symptoms, this study can inform the development of tailored interventions that address the specific needs of vulnerable caregiver populations. Additionally, the findings may contribute to a greater understanding of the complex relationship between cancer, caregiving, and mental health.

Previous research has demonstrated the positive impact of social support on the adjustment of caregivers of cancer patients. Interventions aimed at enhancing social connections may be beneficial in reducing somatization symptoms among this population^35^.

Our study is one of few projects that have focused on the effects of cancer on family members of patients, evaluating somatization as psychologic morbidity and identifying associated parameters influencing the prevalence and severity of these somatic manifestations. However, this study had several limitations. Firstly, the lack of longitudinal follow-up prevented observation of changes in somatic symptoms throughout the patient`s cancer stages. Additionally, our study faced challenges in enrolling a sufficient number of relatives involved with lung cancer patients, potentially limiting the generalizability of our findings. Future research should adopt larger-scale studies with more balanced sample sizes across different cancer types to advance understanding in this area. Furthermore, longitudinal studies conducted over extended periods or at multiple time points would provide valuable insights into the course of somatic symptoms among family members of cancer patients.

## Conclusion

This study raises awareness about the distressing prevalence of somatization, psychological distress that close relatives of cancer patients often struggle with. It underscores the significance of adopting a family-centered approach when treating cancer patients, particularly those with poor prognoses, to enhance the mental and physical well-being of their family members. This approach can help mitigate the psychological distress of the family and elevate the quality of patient care.

## Data Availability

All current study data are available from the corresponding author upon reasonable request.

## Abbreviations

DSM-V: The Diagnostic and Statistical Manual of Mental Disorders 5^TH^ revision
DSM-IV: The Diagnostic and Statistical Manual of Mental Disorders 4^TH^ revision
ICD-10: International Classification of Diseases 10th revision
SOMS-7: The Screening for somatoform symptoms
PHQ-15: The Patient Health Questionnaire
SCL-90: The Symptom Check List
